# Changes in gd T cell function and gut homing receptors following SIV infection of rhesus macaques

**DOI:** 10.1186/1742-4690-9-S2-P271

**Published:** 2012-09-13

**Authors:** I Tuero, M Robert-Guroff

**Affiliations:** 1 National Institutes of Health/National Cancer Institute, Bethesda, MD, USA

## Background

During simian immunodeficiency virus (SIV) infection changes occur in the gd TCR T cell population. The major subset in mucosal tissue, Vd1, becomes prevalent in peripheral blood relative to the Vd2 subset. gd T cells have the ability to expand in vitro and in vivo making them attractive as cytotoxic effectors against infections like SIV and HIV.

## Methods

We studied gut homing receptors and functionality of Vd1 and Vd2 T cells in blood and jejunum of naïve (8 and 3 respectively) and SIV infected (13 and 9 respectively) animals. Vd1+ and Vd2+ T cells were identified by flow cytometry using pan gd TCR-PE and Vd2-FITC mAbs. Intracellular staining for IFN-g and perforin was performed after Phorbol-Myristate-Acetate/Ionomycin stimulation.

## Results

Here, the Vd1 subset in SIV infected macaques was not significantly increased in blood, but in jejunum, Vd1 T cells became predominant compared to Vd2 T cells (p=0.0044) and to the level in blood (p=0.0014). Expansion of the Vd1 subset in jejunum was linked to increased expression of the a4b7 gut homing marker on peripheral blood Vd1 T cells (p=0.033). After stimulation, Vd1 and Vd2 T cells in blood produced both IFN-g and perforin. Higher frequencies of Vd1 T cells produced perforin in SIV+ animals compared to naïve (p=0.0186), while IFN-g producing cell frequencies decreased (p=0.0066). Most Vd1 T cells were CD4-CD8- (DN). Those producing perforin were also mainly DN compared to CD4+ and CD8+ Vd1 T cells (p=0.0156).

## Conclusion

Expansion of Vd1 T cells in the jejunum is associated with increased trafficking from peripheral blood to the mucosal site. This expanded population with cytotoxic potential could contribute to viremia control.

